# Towards Reduced Order Models via Robust Proper Orthogonal Decomposition to capture personalised aortic haemodynamics

**DOI:** 10.1016/j.jbiomech.2023.111759

**Published:** 2023-08-10

**Authors:** Chotirawee Chatpattanasiri, Gaia Franzetti, Mirko Bonfanti, Vanessa Diaz-Zuccarini, Stavroula Balabani

**Affiliations:** a Department of Mechanical Engineering, University College London, London, UK; b Wellcome/EPSRC Centre for Interventional and Surgical Sciences (WEISS), Department of Medical Physics and Biomedical Engineering, University College London, London, UK

**Keywords:** Aortic haemodynamics, Particle image velocimetry, Computational fluid dynamics, Reduced Order Model, Proper orthogonal decomposition, Robust principle component analysis, Patient-specific modelling

## Abstract

Data driven, reduced order modelling has shown promise in tackling the challenges associated with computational and experimental haemodynamic models. In this work, we focus on the use of Reduced Order Models (ROMs) to reconstruct velocity fields in a patient-specific dissected aorta, with the objective being to compare the ROMs obtained from Robust Proper Orthogonal Decomposition (RPOD) to those obtained from the traditional Proper Orthogonal Decomposition (POD). POD and RPOD are applied to *in vitro*, haemodynamic data acquired by Particle Image Velocimetry and compare the decomposed flows to those derived from Computational Fluid Dynamics (CFD) data for the same geometry and flow conditions. In this work, PIV and CFD results act as surrogates for clinical haemodynamic data e.g. MR, helping to demonstrate the potential use of ROMS in real clinical scenarios. The flow is reconstructed using different numbers of POD modes and the flow features obtained throughout the cardiac cycle are compared to the original Full Order Models (FOMs).

Robust Principal Component Analysis (RPCA), the first step of RPOD, has been found to enhance the quality of PIV data, allowing POD to capture most of the kinetic energy of the flow in just two modes similar to the numerical data that are free from measurement noise. The reconstruction errors differ along the cardiac cycle with diastolic flows requiring more modes for accurate reconstruction. In general, modes 1–10 are found sufficient to represent the flow field. The results demonstrate that the coherent structures that characterise this aortic dissection flow are described by the first few POD modes suggesting that it is possible to represent the macroscale behaviour of aortic flow in a low-dimensional space; thus significantly simplifying the problem, and allowing for more computationally efficient flow simulations or machine learning based flow predictions that can pave the way for translation of such models to the clinic.

## 1 Introduction

Restoring flow and functionality is the main objective of clinicians when performing vascular interventions. The ability to visualise biomechanical flows, either by *in vitro* experiments or Computational Fluid Dynamics (CFD), can provide invaluable information for clinical support, disease progression predictions and surgical treatment planning (Bonfanti et al., 2020) by helping design tailored interventions, personalise devices or explore clinical scenarios for future treatment, for example. Application of such tools has been successfully demonstrated in several pathologies, such as aortic dissection (Bonfanti et al., 2020; Franzetti et al., 2022; Stokes et al., 2021, 2023), coronary artery disease (Javadzadegan et al., 2018), valve prosthesis (Hellmeier et al., 2018), aortic aneurysm (Febina et al., 2018) and congenital heart disease (Rigatelli et al., 2021). Both *in vitro* and *in silico* haemodynamic approaches are subject to certain limitations. In CFD for example, a compromise between model accuracy and complexity has often need to be made (Bonfanti et al., 2018). Over-simplifications of the geometrical domain and boundary conditions can lead to non-realistic results. However, increasing the model complexity further complicates the solution, increasing the computational time and often introducing or increasing uncertainty. High computational cost represents a problem for the clinical translation of these numerical models, especially when considering the time-scales of *acute* pathological stages (i.e. days rather than weeks or months) and the limited time available in clinics to make full use of CFD as realistic tool for pre-interventional planning.

To address this problem, Reduced Order Models (ROMs) have been extensively studied to accelerate calculations of fluid dynamic problems (Quarteroni and Rozza, 2007). ROMs replace large-scale problems with less complex ones that can be solved with significantly less time and resources, while maintaining acceptable accuracy. If we were able to extract ROMS from clinical measurement modalities (e.g MR) with a view to use them as input to train fast haemodynamic tools this would address the limitations of traditional CFD and completely transform the use of simulation tools for haemodynamic computations in the clinic. Many methods have been developed to extract ROMs from high-fidelity data, such as Proper Orthogonal Decomposition (POD) (Liang et al., 2002; Brunton and Kutz, 2019; Arzani and Dawson, 2021), Proper Generalised Decomposition (PGD) (Chinesta et al., 2011), Dynamic Mode Decomposition (Arzani and Dawson, 2021; Schmid, 2021), Krylov subspace (Farahbakhsh, 2020), and the recently developed neural network based method, Autoencoder (Wang et al., 2016; Murata et al., 2020; Eivazi et al., 2022; Liang et al., 2020).

Among these methods, POD is arguably the most popular.^1^ POD reduces the dimensionality of a system by projecting it onto a set of orthogonal Reduced Basis (POD modes). It identifies the dominant modes in a flow, breaking it down into large and small-scale structures. With the goal of quantifying different flow regimes and developing computationally-efficient ROMs, POD has been applied to several vascular flow studies, either using numerical CFD data or experimentally-derived velocity fields acquired via Particle Image Velocimetry (PIV). For instance, Kefayati and Poepping (2013) used a combination of PIV and POD to study transitional flows in stenosed silicon models; Byrne et al. (2014) introduced entropy to quantify the flow instability of intracranial aneurysm using POD; Ballarin et al. (2016) developed a framework for the study of haemodynamics in three-dimensional patient-specific configurations of coronary artery by-pass grafts. More recently, Di Labbio and Kadem (2019) compared POD and DMD reconstructions of *in vitro* ventricular flow in a healthy left ventricle and multiple severities of aortic regurgitation, and Han et al. (2020) applied POD to estimate the flow-induced WSS in computational models of abdominal aortic aneurysm.

Robust Proper Orthogonal Decomposition (RPOD) or Robust Principal Component Analysis (RPCA) is an extension of the Proper Orthogonal Decomposition (POD) method, designed to handle noisy or corrupted data commonly found in clinical and experimental datasets (Arzani and Dawson, 2021; Scherl et al., 2020). However, its application in fluid flows, especially in the field of physiological flows, is limited. Previous studies include Scherl et al. (2020), who implemented RPOD filtering in a turbulent channel flow simulation to extract coherent flow structures from the de-noised low-rank matrix, and Baghaie (2019), who applied RPOD to filter out background motion from raw PIV sequences.

In this work, and in the context of using ROMS for clinical application, we will use PIV and CFD results as surrogates for clinical haemodynamic data to demonstrate the potential of the RPOD algorithm when compared to traditional POD for decomposing aortic flow data and constructing ROMs. The RPOD method was applied to the patient-specific aortic, PIV-derived, flow field described in our previous work (Bonfanti et al., 2020; Franzetti et al., 2022). The eigenflows generated are compared to those derived by POD applied to the same data set as well as CFD-derived flow fields for the same geometry. ROMs are then successfully used to reconstruct the original flow fields and their potential for personalised haemodynamic modelling is discussed.

## 2 Materials and methods

**Fig. 1:**
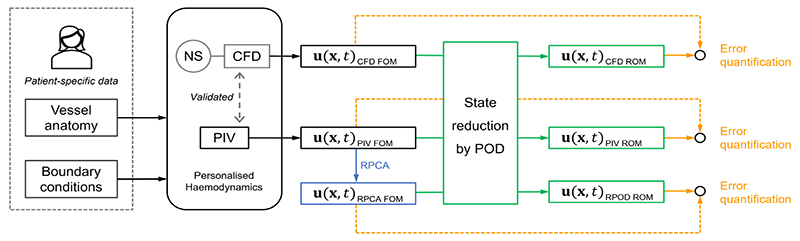
Schematic of the approach followed in this work, comprising four phases. First, the development of Full order models (FOMs), was described in previous works by the authors (Bonfanti et al., 2020; Franzetti et al., 2022) and led to the experimental PIV model and a computational CFD one (black part of the figure). In the second phase, RPCA is applied to the PIV velocity field to create a de-noised RPCA velocity field (blue part of the figure). The third phase involves the creation of ROMs based on the original velocity fields through POD (green part of the figure). In the last phase, the reconstructed velocity fields were compared to their respective original FOMs, the errors involved were assessed (yellow part of the figure). (For interpretation of the references to color in this figure legend, the reader is referred to the web version of this article.)

A schematic of the approach followed in this work is shown in Fig. 1. We have previously characterised and fully validated the flow in a patient-specific dissected aorta both experimentally (using PIV) and numerically (using CFD) (Franzetti et al., 2022; Bonfanti et al., 2020) (black part of the figure). These datasets comprise the Full Order Models. The RPCA algorithm was applied to the PIV velocity field to create a de-noised dataset, which we call RPCA velocity field (blue part of the figure).

The state reduction of the problem was then achieved by projecting the CFD, PIV, and RPCA velocity fields onto their POD bases to reduce the dimensionality of the problem (Galerkin projection). ROMs were identified, ROM-derived flow fields were reconstructed (green part of the figure) and compared to the FOMs. The errors introduced when considering a lower dimensional model were assessed (Yellow part of the figure).

### 2.1 Patient-specific model

The study is based on clinical data acquired from an adult male with a Type B aortic dissection, a pathology that occurs when a tear in the vessel wall allows blood to flow within the layers of the aorta, leading to the formation of two separate flow-channels, the true and the false lumen. The dataset was acquired as part of an ethically-approved protocol at the Leeds General Infirmary (NHS Health Research Authority, ref: 12/YH/0551; Leeds Teaching Hospitals NHS Trust, ref: 788/RADRES/16), and appropriate concent was obtained from the patient. The aortic model was created from the patient CT scans using a semi-automated segmentation tool based on thresholding operations, implemented in ScanIP (Synopsys, Mountain View, CA, USA). It includes one inlet and four outlets: the brachiocephalic trunk (BT), left common carotid (LCC), left subclavian artery (LSA), and descending aorta (DA). A rigid, transparent phantom was manufactured by 3D printing technology (Materialise, Belgium) to enable the flow field measurements described below.

### 2.2 Experimental setup and PIV measurements

**Fig. 2:**
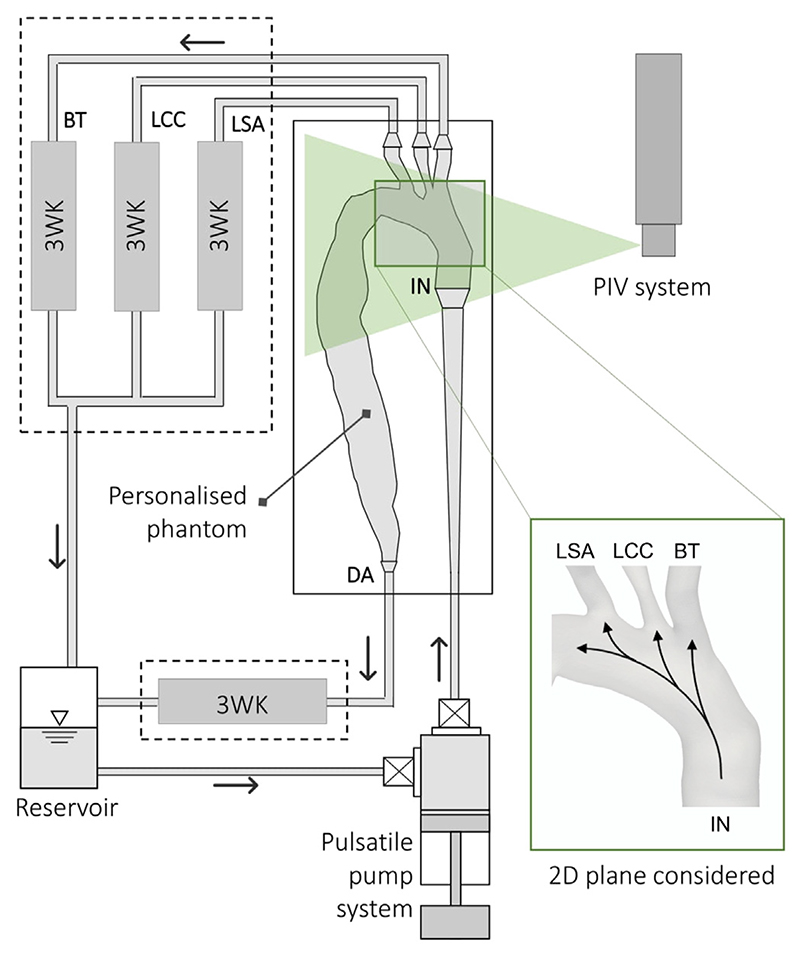
Schematic of the experimental setup. The rig comprises a pulsatile pump system to provide the patient-specific inlet flow rate into the aortic phantom; four 3-elements Windkessel models (3WKs) – one for each of the outlets: brachiocephalic trunk (BT), left common carotid (LCC), left subclavian artery (LSA), and descending aorta (DA) – and an aortic reservoir. The 2D plane where the PIV acquisitions considered in this work were performed is also represented. Superimposed arrows qualitatively indicate the direction of the flow during systole.

The phantom was connected to a custom-made pulsatile flow circuit which comprised a computer controlled pulsatile pump and left ventricle simulator, tunable 3-element Windkessel (3WKs) model at each aortic outlet (Fig. 2) and an atrial reservoir (Franzetti et al., 2019). The mock loop components were informed by clinical data to reproduce personalised, accurate haemodynamics (Franzetti et al., 2022). A blood mimicking fluid comprising a potassium thiocyanate (KSCN) water solution (63% by weight) was used, matching the refractive index of the phantom. Patient-specific flow and pressure waveforms were introduced at the inlets and outlets of the aortic model as illustrated in Bonfanti et al. (2020) and Franzetti et al. (2022). To perform the PIV measurements, the flow was seeded with fluorescent microparticles with a mean diameter of 10 μm, injected into the flow upstream of the phantom and allowed to disperse uniformly within the aortic model. The flow was illuminated by a pulsed Nd:YAG laser (Litron Lasers, Bernoulli, UK) emitting 532 nm wavelength light. Particle image pairs were acquired with a CCD camera (Imperx, USA) at a sampling rate of 22 Hz (the pulsatile flow has a frequency of 1.2 Hz) with a resolution of 4000 × 3000 pixels with a time interval of 1 ms. 10 cardiac cycles were recorded.^2^ Velocity fields were generated using the Fast Fourier transform based cross-correlation algorithm, implemented with a three-pass technique starting with an interrogation area of 64 × 64 pixels and ending with an area of 32 × 32 pixels, overlapping by 50%. Lastly, post-processing was performed using custom developed MATLAB (MathWorks Inc., USA) functions. The measurement error, estimated from mass conservation, is 5.32% (Franzetti et al., 2022).

Details about the components of the mock circulatory loop and the experimental procedures can be found in our previous work (Franzetti et al., 2019, 2022). Here, the PIV-derived velocities obtained on a cross-sectional plane of the aortic arch (shown in Fig. 2), consisting of 10 cardiac cycles with 18 snapshots per cycle, i.e. 180 snapshots in total, were used. This minimises the risk of bias introduced by using a single cycle, and better represents flow behaviour.

### 2.3 PIV data enhancement by RPCA

Robust Principal Component Analysis (RPCA) or Robust Proper Orthogonal Decomposition (RPOD) is an extension of PCA or POD that separate an original data matrix (**U**) into a sparse noise matrix (**S**) and a low-rank matrix containing coherent information (**L**) using the following equation:

(1) U=L+S.

RPCA is known for its ability to handle noisy data (Scherl et al., 2020; Arzani and Dawson, 2021; Candès et al., 2011; Brunton and Kutz, 2019) and is a good candidate to enhance the PIV data prior to further analysis. The implementation of RPCA involves solving a constrained minimisation equation:

(2) $$\underset{L,S}{\min}\text{(rank}(L)+{\left\|S\right\|}_{0}\text{)}\;\text{subject}\;\text{to}\;U=L+S$$

where ∥**S**∥_0_ represents the zero norm of **S**, which is the summation of non-zero elements in **S**. A convex relaxation (2) form of the problem is used:

(3) $$\underset{L,S}{\min}\left(\|L{\|}_{*}+\lambda_{0}\|S{\|}_{1}\right)\;\text{subject}\;\text{to}\;U=L+S$$

where ∥**L**∥_*_ represents the nuclear norm of **L**, which is the summation of all the singular values of **L**, and ∥**S**∥_1_ denotes the first norm of **S**. *λ*_0_ is a hyperparameter introduced as part of the relaxation given by $\lambda_{0}=\lambda_{1}/\sqrt{\max(m,n)}$ where *m × n* is the dimension of **U** and *λ*_1_ = 1 in the original paper (Candès et al., 2011). Scherl et al. (2020) suggested that *λ*_1_ can also be used as a tuning parameter: high *λ*_1_ yields high sparsity of **S**, and low *λ*_1_ gives low rank of **L**. For simplicity, *λ*_1_ is kept at 1 in this study. Eq. (3) can be solved using the Augmented Lagrange Multiplier (ALM) algorithm together with the Alternating Direction Method (ADM) (Brunton and Kutz, 2019).

RPCA is used here as a de-noising tool to improve the PIV velocity field and thus is not applied to the CFD data. It is important to note that, because RPCA distinguishes outliers from coherent data, it may struggle when data points are scarce, blurring the difference between outliers and coherent information. Therefore, all acquired PIV snapshots (180) were used.

The term ‘RPCA velocity field’ is used to denote the PIV velocity field that has been de-noised by the RPCA process, whereas the term ‘RPOD’ refers to the implementation of POD after RPCA.

### 2.4 Numerical simulation

The CFD data is taken from a previous study by our group (Bonfanti et al., 2020). A Reynolds-averaged Navier–Stokes (RANS) model was employed in this study to match the experimental inlet conditions since it is very difficult to achieve truly laminar flow in experiments. While RANS models are known to present certain limitations, such as not capturing unsteady turbulence fluctuations and assuming fully turbulent flow, they offer a good compromise between accuracy and computational efficiency in comparison to more resource-intensive methods like Large Eddy Simulation (LES) and Direct Numerical Simulation (DNS); they were hence selected for this study and showed good agreement with the experimental data (Bonfanti et al., 2020). ANSYS-CFX 19.0 (ANSYS, USA) was used to solve the 3D incompressible Navier–Stokes and continuity equations, simulating the flow conditions of the PIV experiment. The vessel geometry is the same as the one used to make the phantom, with the walls assumed rigid. All boundary conditions are set to match those from the experiments. The experimental inlet flow rate waveform is imposed with a flat velocity profile. 3WKs models are coupled at the outlets. The fluid is assumed to be Newtonian, with the properties of the KSCN solution. The shear stress transport (SST) turbulence model is chosen with the turbulence intensity of 1% prescribed at the inlet.

The simulations run for 3 cardiac cycles and the first two cycles were excluded from the analysis as they contain transient behaviour influenced by the initial conditions and numerical setup. Unlike in the flow field obtained from PIV experiments, in the case of CFD, once the simulations have converged to the periodic steady state, every cycle is identical. Therefore, a single cycle is used in our study as is commonly done in most patient specific simulations (Bonfanti et al., 2020; Stokes et al., 2023). The flow field on the 2D aortic arch plane corresponding to the experimental one used here was exported to MATLAB via CFD-Post (ANSYS).

### 2.5 Proper orthogonal decomposition

The POD method decomposes the flow into a set of *modes* arranged depending on their energy content. The higher energy modes represent the coherent structures in the flow; as a result POD has been applied widely to turbulent flows to extract dominant structures. A detailed description of POD can be found in Berkooz et al. (1993) and in the textbook by Brunton and Kutz (2019). Only a brief overview is provided here.

POD is implemented using the method of *snapshots*. Consider a 2D velocity field of *n = N_x_ × N_y_* spatial velocity vectors (*u, v*) on a Cartesian grid *x, y* and a total number of *m* instantaneous velocity fields or *snapshots*. POD decomposes the fluctuating part of the velocity field **u**′(*x, y, t*) into a set of spatial functions **Φ**_*i*_(*x, y*), called the *POD modes*, weighted by time-dependent coefficients *a_i_*(*t*) so that:

(4) $$u^{\prime }(x,y,t)=\overset{N}{\underset{i=1}{\sum^{\text{​}}}}a_{i}(t)\Phi_{i}(x,y)$$

where *i* denotes the mode number, and *N* denotes the total number of modes.^3^

To perform the decomposition (Eq. (4)), the time-averaged velocity $\bar{u(x,y)}$ is first subtracted from each instantaneous velocity field, obtaining a set of *m* fluctuating velocity fields **u**′(*x, y, t*). The dataset is then rearranged in a 2*n × m snapshot matrix* **U**:

(5) $$U=\left(\begin{array}{cccc} u_{1,1}^{\prime } & u_{1,2}^{\prime } & \cdots & u_{1,m}^{\prime }\\ \vdots & \vdots & \ddots & \vdots \\ u_{n,1}^{\prime } & u_{n,2}^{\prime } & \cdots & u_{n,m}^{\prime }\\ v_{1,1}^{\prime } & v_{1,2}^{\prime } & \cdots & v_{1,m}^{\prime }\\ \vdots & \vdots & \ddots & \vdots \\ v_{n,1}^{\prime } & v_{n,2}^{\prime } & \cdots & v_{n,m}^{\prime } \end{array}\right)$$

and Singular Value Decomposition (SVD) is applied:

(6) $$U={\Phi \Sigma \Psi }^{*}$$

where **Φ** and **Ψ** are the left and right singular vectors of **U**, respectively and Ψ* is the conjugate transpose of **Ψ**. The singular matrix (**Σ**) contains the singular values (*σ_i_*) of **U** which rank in descending order, and are directly linked to the portion of kinetic energy (*λ_i_*) contained in the POD modes (**Φ**_*i*_), i.e. $\lambda_{i}=\sigma_{i}^{2}$ Therefore, POD modes are ranked according to their energy content, with the first mode having the highest energy, and the last the lowest. The energy fraction of the *i*th mode *E_i_*, is defined as

(7) $$E_{i}=\frac{\lambda_{i}}{\overset{N}{\underset{i=1}{\sum^{\text{​}}}}\lambda_{i}}$$

The temporal POD coefficients can be obtained by projecting **U** onto **Φ**_*i*_:

(8) $$a_{i}(t)=\Phi_{i}^{*}U$$

The total number of POD modes (*N*) is the rank of **U**, which is usually equal to the number of snapshots considered. The PIV data contains 10 cardiac cycles with 18 instants per cycle, leading to 180 modes in total. For the CFD data, the flow field considered consists of only one cardiac cycle with 165 snapshots, resulting in 165 modes in total. The RPCA velocity field, on the other hand, consists of 180 snapshots since it was generated from PIV data, but consists of only 35 modes. This is because the RPCA process seeks to obtain a low-rank representation of the original data.

### 2.6 Reduced order model

To generate a low dimensional representation of the aortic flow under consideration, Eq. (9) can be applied to reconstruct the flow field from the POD modes and the mean velocity $\bar{u(x,y)}$ as:

(9) $$u(x,y,t)=\bar{u(x,y)}+\overset{r}{\underset{i=1}{\sum^{\text{​}}}}a_{i}(t)\Phi_{i}(x,y)$$

where *r* denotes the number of modes included in the ROM. When setting *r = N*, where *N* denotes the total number of POD modes, Eq. (9) yields FOM. Equivalently, the reconstructed snapshot matrix **U**_*r*_ can be calculated from:

(10) $$U_{r}=\Phi_{r}\Sigma_{r}\Psi_{r}^{*}$$

with **Φ**_*r*_, **Σ**_*r*_, and **Ψ**_*r*_ are the truncated versions of **Φ**, **Σ**, and **Ψ**, respectively. The reconstruction error is defined as:

(11) $$\varepsilon =\frac{\overset{m}{\underset{j=1}{\sum^{\text{​}}}}\overset{2n}{\underset{i=1}{\sum^{\text{​}}}}\left|U(i,j)-U_{r}(i,j)\right|}{\overset{m}{\underset{j=1}{\sum^{\text{​}}}}\overset{2n}{\underset{i=1}{\sum^{\text{​}}}}|U(i,j)|}\times 100\text{\%}$$

The POD spatial structures and temporal coefficients were used to characterise specific flow features — *coherent structures* — in the pulsatile, aortic dissection flow, separating the periodic and random fluctuating structures from the mean flow. The spatial structures **Φ**_*i*_ for the relevant modes were analysed by plotting the velocity fields. Then, the temporal characteristics of the flow were investigated by analysing the temporal coefficients of the most energetic POD modes in both the time and frequency domains.

Lastly, flow field reconstructions from the ROMs were performed according to Eq. (9). First **u**(*x, y, t*) was reconstructed using all the modes to verify the accuracy of the mathematical calculations. Then, they were reconstructed using only a selected number of modes (i.e. **Φ**1–2, **Φ**1–5, and **Φ**1–10) and the solution was compared to the original velocity fields, at different instants of the cardiac cycle, to quantify the differences.

## 3 Results and discussion

### 3.1 Kinetic energy distribution

Table 1 lists the cumulative kinetic energy contents of the POD and RPOD modes derived from the PIV data (PIV POD, PIV RPOD respectively) compared to those derived from the CFD data (CFD POD). The energy contained in the ROMs is expressed as a percentage of the total kinetic energy in their respective FOMs, i.e. the total energy of the original or filtered PIV and CFD velocity fields respectively. While more than 90% of the kinetic energy is reached within the first 2 modes for the PIV RPOD and CFD POD data, it takes 10 modes for the PIV POD to capture that amount of energy. Similarly, Table 1 also shows that the reconstruction errors from PIV RPOD and CFD POD converge to zero faster than the ones from PIV POD.

This is not surprising as the PIV data are subject to measurement noise. In contrast, the CFD velocity field was derived from numerical computations, no measurement errors are involved. Additionally, the use of a RANS model in CFD inherently ignores turbulent fluctuations, resulting in much cleaner data compared to PIV.

The RPCA process denoises the PIV data resulting in higher cumulative energy in the first two and ten modes compared to that from PIV data alone. The energy content of each mode becomes closer to that derived from the CFD data.

### 3.2 POD structures and temporal coefficients

**Fig. 3:**
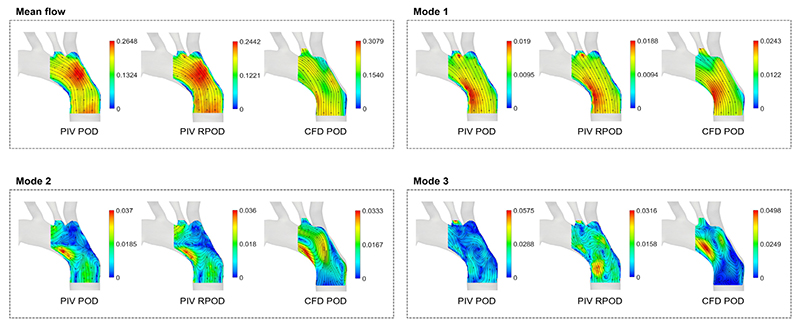
The mean velocity field and the first three POD structures (**Φ**1–3) obtained from the PIV, RPCA, and CFD velocity fields, respectively. Super-imposed streamlines were used for illustration purposes, they do not convey any information on temporal variations. Please note that the scales of the contours are different.

Selected POD structures (**Φ**1–3) are shown in Fig. 3 compared with the mean flow. All the first POD structures (**Φ**1) show organised motion in the same direction as the mean flow which reflects the flow at the peak systolic phase (Franzetti et al., 2022). The structures extracted from PIV POD and PIV RPOD appear to be almost identical as they represent the most energetic flow features; they slightly differ from the CFD POD ones due to the differences between the measured and computed velocity fields discussed in our previous work (Bonfanti et al., 2020).

Modes 2 and 3 (**Φ**2–3) exhibit more complex flow patterns characterised by re-circulation regions. As for **Φ**2, a high magnitude region can be seen at the inner side of the arch which reflects the flow pattern in diastole.

**Fig. 4:**
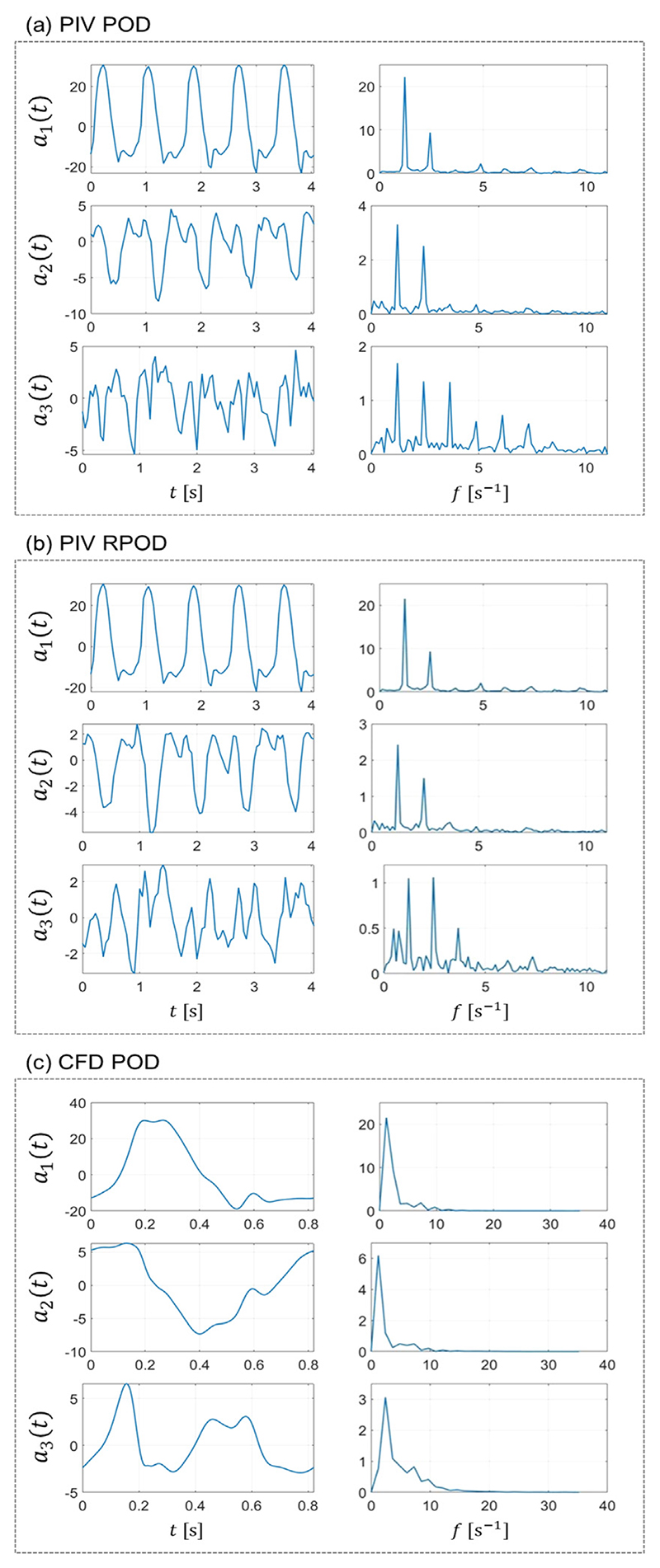
The first three POD temporal coefficients (*a*_1_, *a*_2_, and *a*_3_) from (a) PIV POD, (b) PIV RPOD, and (c) CFD POD presented in (left) time domain and (right) frequency domain.

The temporal coefficients (*a_i_*) of the first three POD modes are shown in Fig. 4 in the time (left column) and frequency domain (right column), respectively. The coefficients exhibit periodic characteristics in agreement with the literature (Kefayati and Poepping, 2013). The first temporal coefficient essentially reflects the shape of the patient-specific inlet flow waveform manifesting with a dominant peak in the spectra at the frequency of the cardiac cycle (f = 73.2 bpm = 1.22 Hz). As the number of modes increases, the temporal coefficients show more complicated patterns characterised by higher frequency oscillations. A second harmonic (2.44 Hz) is evident on the frequency spectra of the experimentally derived modes. This behaviour may be related to velocity fluctuations due to transitional flow. This behaviour is absent from the numerical POD coefficients which consist of one single cardiac cycle only, hence oscillating at the cardiac cycle frequency only.

### 3.3 Flow reconstructions

**Fig. 5:**
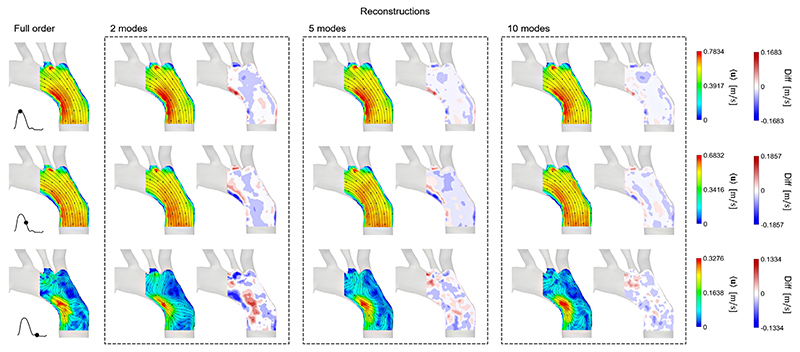
PIV POD, a POD reconstruction of PIV velocity field using **Φ**1–2, **Φ**1–5, and **Φ**1–10. The FOM and reconstructed flow fields are visualised at three instances of the cardiac: peak systole, deceleration, and diastole. Contours showing the difference in velocity magnitude between the FOM and reconstructed flow fields are shown side by side with the reconstructed velocity field.

**Fig. 6:**
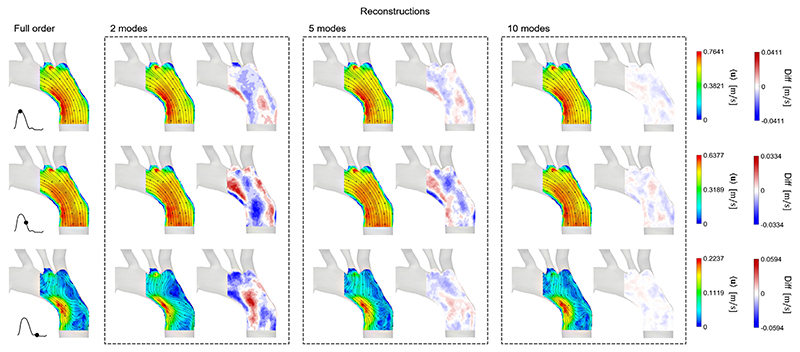
PIV RPOD, a POD reconstruction of RPCA velocity field using **Φ**1–2, **Φ**1–5, and **Φ**1–10. The FOM and reconstructed flow fields are visualised at three instances of the cardiac: peak systole, deceleration, and diastole. Contours showing the difference in velocity magnitude between the FOM and reconstructed flow fields are shown side by side with the reconstructed velocity field.

**Fig. 7:**
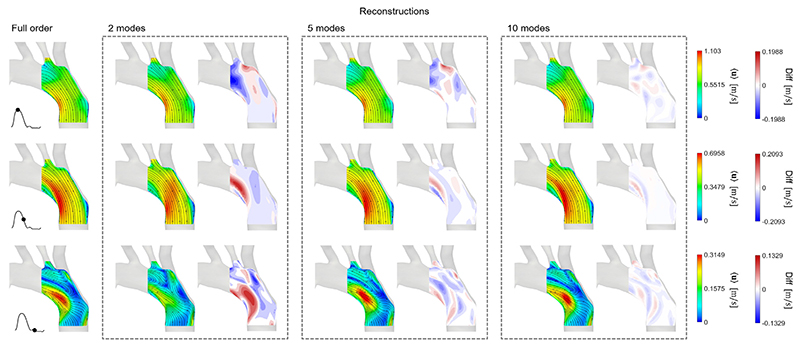
CFD POD, a POD reconstruction of CFD velocity field using **Φ**1–2, **Φ**1–5, and **Φ**1–10. The FOM and reconstructed flow fields are visualised at three instances of the cardiac: peak systole, deceleration, and diastole. Contours showing the difference in velocity magnitude between the FOM and reconstructed flow fields are shown side by side with the reconstructed velocity field.

Figs. 5–7 show the comparison between the FOMs at three instants of the cardiac cycle (peak systole, deceleration and diastole), and the reconstructed velocity fields of ROMs using **Φ**1–2, **Φ**1–5 and **Φ**1–10. The figure also shows contours of the differences in velocity magnitude between the original and reconstructed flow fields. The reconstruction errors are shown in Table 1. As expected, in all cases, the more POD/RPOD modes are included in the reconstruction, the lower the error.

The reconstructed flow fields from the PIV POD in Fig. 5 show that good agreement can be achieved even using the first 2 POD modes at peak systole and in the descending part of the flow curve, where the flow follows relatively organised uni-directional patterns, with some differences occurring in near-wall re-circulation regions. However, more significant discrepancies are observed in diastole, both in terms of velocity magnitude, distribution and the flow directions indicated by the streamlines. Such differences are to be expected because, at diastole, the flow contains smaller vortical structures and a more complex flow field. To fully reconstruct these structures, a higher number of modes should be included. 10 modes appear to be sufficient to reconstruct the flow at diastole accurately. When reconstructing the flow with modes **Φ**1–10, the maximum absolute difference is about 0.09–0.10 m/s and occurs at systole.

Similar reconstructed velocity fields are generally obtained from the PIV RPOD in Fig. 6. The only noticeable difference is at diastole, where the RPCA velocity field exhibits lower velocity magnitudes compared to the PIV-derived one, due to its filtering action. The RPCA velocity magnitude ranges from 0 to 0.2237 m/s, while the original flow field from 0 to 0.3276 m/s at diastole. When using the same number of modes to reconstruct the flow field, PIV RPOD has a much lower reconstruction error than PIV POD. The maximum absolute error when using 10 modes for the flow reconstruction is only 0.007–0.008 m/s, i.e. 10 times lower than that in PIV POD.

Finally, Fig. 7 shows that the flow reconstructions from the CFD POD analysis share similar important qualities with the experimental ones; namely that the first 2 modes are able to reconstruct the velocity field accurately at peak systole and deceleration phase, with the errors decreasing rapidly when more modes are included. The difference contour plots show less scatter compared to the PIV derived ones due to high spatial resolution of the numerical data. A maximum difference of around 0.05–0.06 m/s is found when reconstructing the flow fields using 10 modes; this is smaller than the errors in PIV POD but slightly higher than the case of PIV RPOD.

It is important to highlight that all the errors reported above are reconstruction errors. They are obtained by comparing the velocity fields from ROMs to those of their respective FOMs. These errors are not from comparisons among the PIV velocity fields, RPCA velocity fields, and CFD velocity fields (see Appendix A). Thus, they cannot be interpreted as such.

### 3.4 Towards personalised ROMs

The use of the RPCA algorithm in this study successfully filtered out high-frequency noise in the PIV data (see Fig. 4), improving the performance of ROMs extracted from the data. In the context of dimensionality reduction, the application of RPCA to PIV data leads to representations in a lower-dimensional space compared to the original PIV data. This feature of RPCA might be helpful when analysing the images from MR data for example, which would help pave the way towards the use of these numerical tools in a clinical setting.

However, RPOD may over-filter certain parts of the flow field as can be seen during diastole (Figs. 5–7 bottom row). While the velocity magnitude of the PIV and CFD derived fields are in the same range, it is significantly lower (by about 30%) for the RPCA derived one. This shows that the algorithm may filter out some important flow features and if implemented in a real clinical pathway, might require careful consideration. This over-filtering issue can be addressed by increasing the value of the tuning parameter *λ*_1_. However, too high a value of *λ*_1_ can lead to the presence of noise in the filtered data. Therefore, the main challenge involved in the application of RPCA is to find the optimal value of *λ*_1_ that appropriately filters out the unwanted motion while preserving the relevant ones. Future work can focus on fine tuning *λ*_1_ for a given dataset or exploring the integration of a physics-informed regularisation term into the RPCA framework to preserve the velocity signal while still reducing noise.

Nevertheless, the enhancement of *in vitro*, experimental data using RPCA demonstrated here, suggests that such methods could potentially be applied to *in vivo* data, such as 4D flow MRI, to improve their quality, making them more amenable to computational modelling and flow reconstruction (Bakhshinejad et al., 2017; Fathi et al., 2018; Töger et al., 2020) for patient-specific studies. In addition, the ability of the RPCA algorithm to effectively clean data may also result in ROMs constructed from RPOD having to include fewer modes, leading to faster computations in their subsequent applications, which is an important feature if these techniques were to be incorporated in real clinical pathways.

This work also demonstrates that it is possible to represent the behaviour of complex, pathological aortic flows using ROMs consisting of only the first few POD/RPOD modes, which shows promise in the development of more computationally efficient models to support clinical decision-making. Examples include the works of Chang et al. (2017) who developed a computationally efficient ROM to study the flow patterns and the WSS distribution in simplified models of an abdominal aortic aneurysm, and Buoso et al. (2019), who developed ROMs of blood flow for non-invasive functional evaluation of the pressure drop in coronary artery disease using parameterised POD. ROMs may also possess properties that can serve as supplementary haemodynamic indices. For example, by monitoring the temporal evolution of energy distribution, it may be possible to track the progression of some cardiovascular diseases or even vascular remodelling. Moreover, the energy fraction associated with higher-order POD/RPOD modes may contain information that can be used to fine-tune turbulence parameters when modelling vascular flows.

Finally, ROMs can also be combined with rapidly evolving machine learning tools to allow for *optimisation* and *design* in fluid mechanics, moving towards real-time modelling. This would allow, for instance, the study of a wide range of parameters for a given vascular pathology (e.g. increasing or decreasing the level of stenosis on coronary disease or coarctations) and to analyse the consequences on the flow and pressure fields, which could serve as an initial step to investigate patient-specific pre-interventional options (Siena et al., 2023; Pajaziti et al., 2023; Liang et al., 2020).

## 4 Conclusions

The time-dependent flow in an aortic model, measured by PIV, was enhanced by RPCA and decomposed by means of POD to create ROMs. The decomposed flows were compared against those from numerical data obtained for the same patient-specific conditions. The first two modes derived from RPOD capture more than 90% of the kinetic energy, in agreement with the corresponding CFD derived ROMs.

The large and small-scale structures within the flow, corresponding to more or less energetic modes, were evaluated and described by means of POD/RPOD spatial structures and POD/RPOD temporal coefficients. By combining only the most energetic modes to represent the flow, it was shown that complex, time-dependent haemodynamic data can be represented with simpler low-dimensional models based on a small number of spatial modes. This combined with the strong reconstruction performance of RPOD, illustrates the potential of the approach to enhance the quality of measurements and to develop more computationally efficient models for clinical application.

## Supplementary Material

Video S1

Appendix

## Figures and Tables

**Table 1:** Percentage of Kinetic energy captured and reconstruction error of different groups of POD modes calculated from PIV velocity field, RPCA velocity field, and CFD velocity field.

*Modes*	PIV POD		PIV RPOD		CFD POD	
	Energy(%)	Error(%)	Energy(%)	Error(%)	Energy(%)	Error(%)
1–2	86.33	34.18	98.15	13.10	95.52	19.54
1–5	89.57	30.16	99.37	7.85	98.77	10.18
1–10	91.72	27.06	99.78	4.60	99.72	4.81
all	~100	~0	~100	~0	~100	~0

## Footnotes

1 POD is mathematically equivalent to Principal Component Analysis (PCA) (Liang et al., 2002).

2 The flow converged with less than 1.6% cycle-to-cycle variation (Bonfanti et al., 2020; Franzetti et al., 2022).

3 Note that **u**′ in Eq. (4) includes both the periodic variation around the mean and the turbulence fluctuation.
