# Supplementary material for: Towards Reduced Order Models via Robust Proper Orthogonal Decomposition to capture personalised aortic haemodynamics
Source: J Biomech. Author manuscript; Available in PMC 2024 Mar 7. (PMC7615718; doi:10.1016/j.jbiomech.2023.111759)
Supplement: Appendix [file EMS194351-supplement-Appendix.pdf]

Appendix A. Comparison of velocity fields between CFD and PIV before and after RPCA

The comparisons of PIV, RPCA, and CFD velocity fields is shown in Fig. A.1. Fig. A.1a shows the velocity magnitude of the mean flow in the three velocity fields with four straight lines. A closer comparison

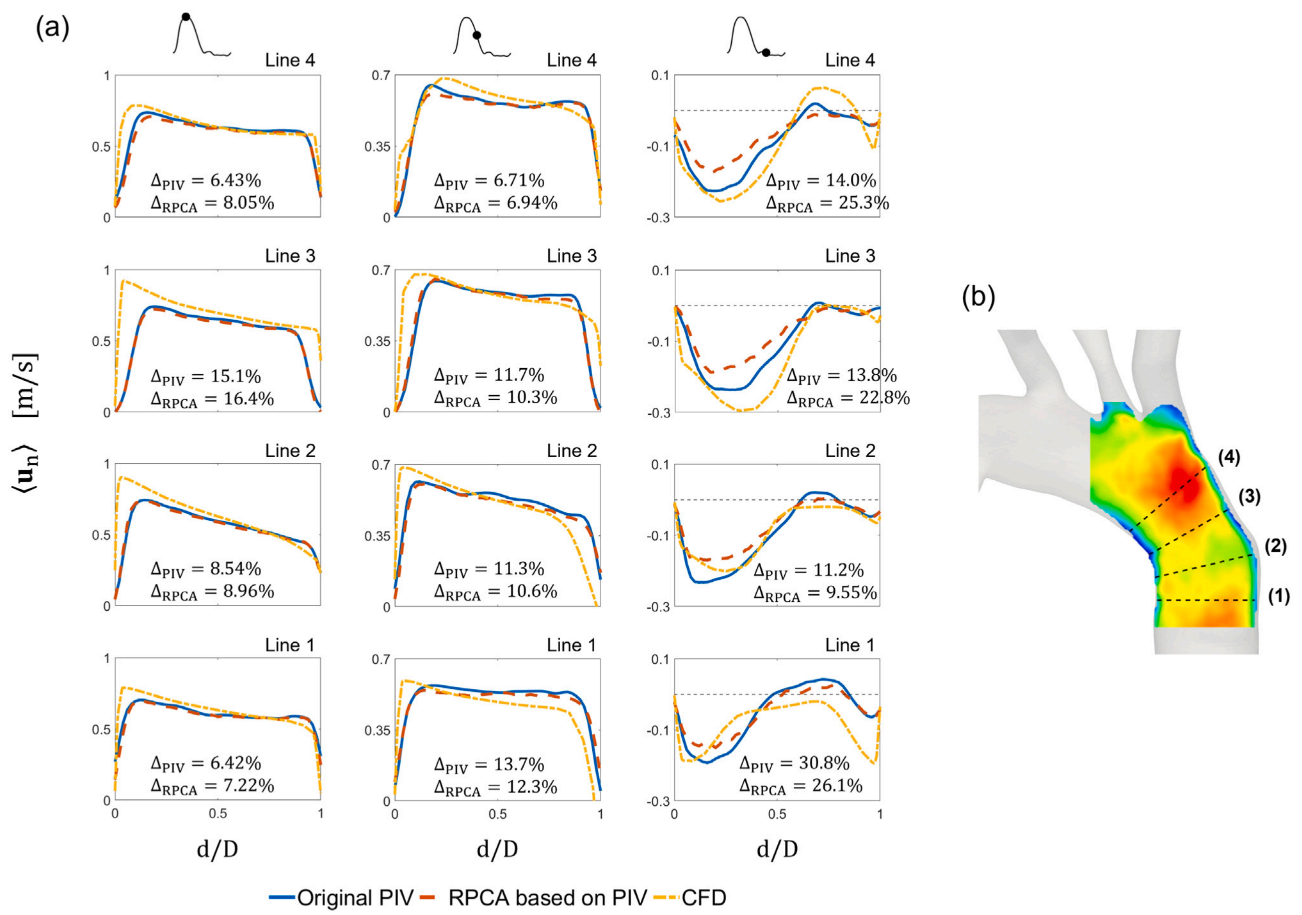

Fig. A.1. (a) Comparison of axial velocity profiles obtained from experimental PIV data before RPCA, after RPCA, and CFD data. (b) Four lines across the aortic arch where the velocity profiles were measured.

between the three velocity fields is provided in Fig. A.1b by plotting the axial velocity profiles ($\langle \mathbf{u}_n \rangle$) over those four selected lines.

The percentage difference is calculated in the same way as in Bonfanti et al. (2020):

$$\Delta = \frac{1}{N} \sum_{i=1}^{N} \frac{\left| u^{e}_{n,j} - u^{c}_{n,j} \right|}{\max_j \left| u^{c}_{n,j} \right|} \tag{12}$$

where $u^{c}_{n,i}$ is the CFD velocity profile. When calculating Δ_{PIV}, $u^{e}_{n,i}$ is the PIV velocity profile, and when calculating Δ_{RPCA}, $u^{e}_{n,i}$ is the RPCA velocity profile. It should be noted that the percentage differences are not exactly the same as those reported in Bonfanti et al. (2020) because there might be discrepancies in the locations of the four lines.

As shown from the above (Fig. A.1b), RPCA may not always bring the PIV and CFD closer together in terms of the actual velocity values due to the over-filtering behaviour discussed in Section 3.4. However, RPCA leads to PIV derived ROMs that capture the same percentage of energy as CFD ones when reconstructing with the same number of modes (Table 1). To help visualise the effect of RPCA on PIV data, comparison can be found in the video file attached (or here).

Appendix B. Normalised POD/RPOD coefficients

The relation between the first coefficients a_i was investigated by plotting the space (a^*_1, a^*_2), calculated as:[4]

$$\tilde{a}_i = \frac{a_i}{\sqrt{2\lambda_i}} \tag{13}$$

and normalise as

$$a^*_i = \frac{2(\tilde{a}_i - \min(\tilde{a}_i))}{(\max(\tilde{a}_i) - \min(\tilde{a}_i))} - 1 \tag{14}$$

The normalised temporal coefficients a^*_1, a^*_2 and a^*_3, are plotted against each other in Fig. B.1. The phase-averaged coefficients for the experimental data are also indicated in blue. They exhibit organised, closed-loop structures, indicating periodicity similar to the CFD data.

The 2D plots of a^*_1 and a^*_2 in Fig. B.1 do not show a clear circular or elliptic pattern, implying that the first two POD modes do not form a pair. The plots also indicate the same behaviour for the first two PIV POD and PIV RPOD coefficients which slightly differs from the CFD POD ones.

An interesting observation arises when investigating the relation amongst the first three coefficients (a^*_1, a^*_2 and a^*_3). The plots on the right side of Fig. B.1a–c show a more complex organisation amongst these modes. This behaviour highlights the interdependent relationships and energy transfer between the first three modes and may correspond to energy transfer amongst different periodic structures (with different energy contents and frequency profiles) within the flow field. A similar *'triadic interaction'* has been reported by Gabelle et al. (2017) in a stirred tank flow (who attributed the behaviour to non-linear interactions between the modes), and by Lacassagne et al. (2021) in an oscillating grid flow.

[4] This approach has been used in different studies, for example see Imomoh et al. (2010) and Ducci et al. (2008).

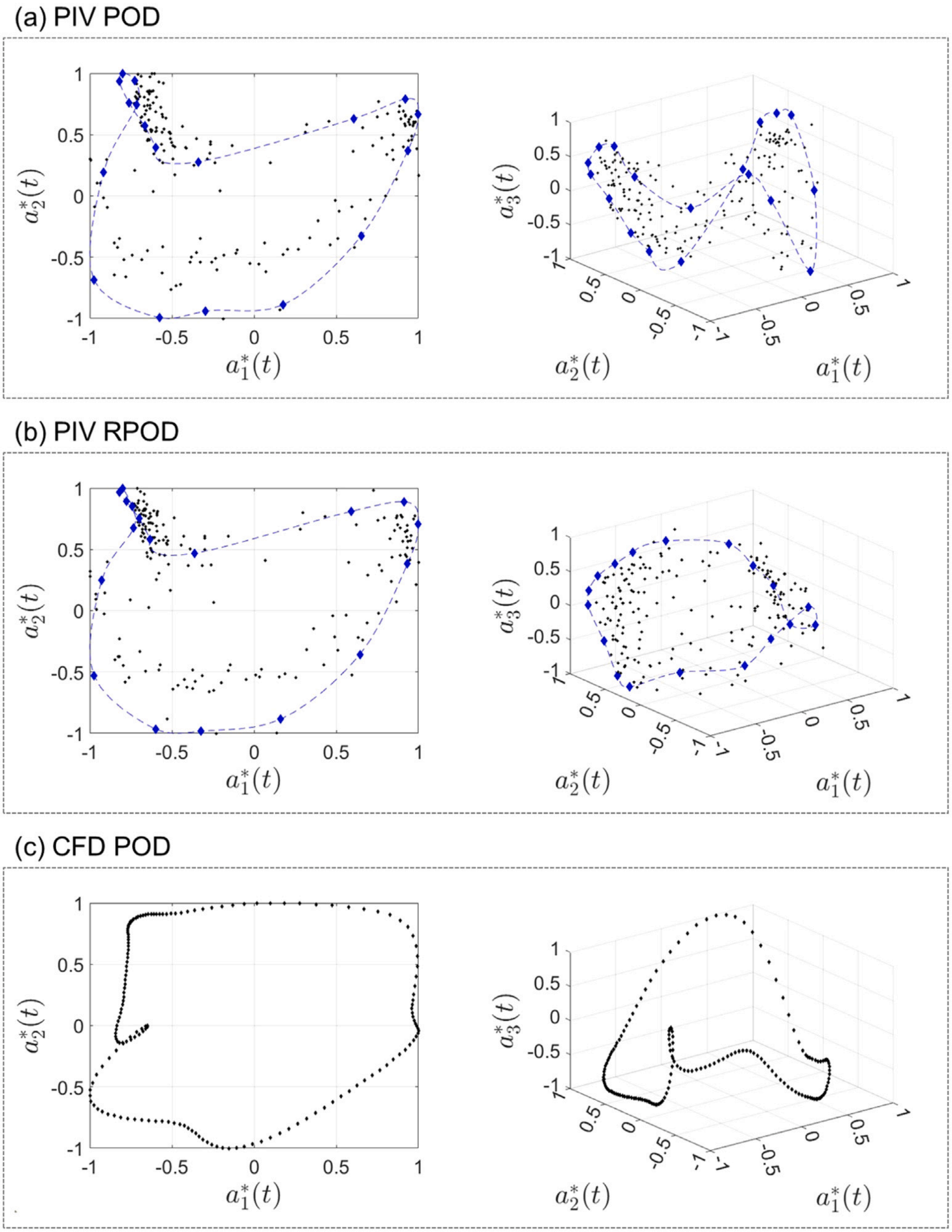

Fig. B.1. Scatter plots of normalised POD coefficients (left) a_1^* and a_2^* and (right) a_1^*, a_2^* and a_3^* computed from (a) PIV POD, (b) PIV RPOD, and (c) CFD POD. The black points represent the coefficients for all the modes, whilst the blue ones represent the phase-averaged POD modes. The blue lines connect the phase-averaged coefficients for better visualisation. (For interpretation of the references to color in this figure legend, the reader is referred to the web version of this article.)
